# Butane-1,4-diammonium bis(perchlorate)

**DOI:** 10.1107/S1600536811012025

**Published:** 2011-04-07

**Authors:** Charmaine Arderne, Gert J. Kruger

**Affiliations:** a Department of Chemistry, University of Johannesburg, PO Box 524, Auckland Park, Johannesburg, 2006, South Africa

## Abstract

The butane-1,4-diammonium cation of the title compound, C_4_H_14_N_2_^2+^·2ClO_4_^−^, lies on a special position of site symmetry 2/*m*, whereas the perchlorate anion is located on a crystallographic mirror plane. An intricate three-dimensional hydrogen-bonding network exists in the crystal structure with each H atom of the ammonium group exhibiting bifurcated inter­actions to the perchlorate anion. Complex hydrogen-bonded ring and chain motifs are also evident, in particular a 50-membered ring with graph-set notation *R* ^10^_10_(50) is identified.

## Related literature

For related structural studies of butane-1,4-diammonium salts, see: van Blerk & Kruger (2007 ▶); Lemmerer & Billing (2006 ▶); Gabro *et al.* (2009 ▶). For hydrogen-bond motifs, see: Bernstein *et al.* (1995 ▶).
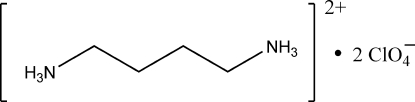


## Experimental

### 

#### Crystal data

C_4_H_14_N_2_^2+^·2ClO_4_^−^
*M* *_r_* = 289.07
Monoclinic, 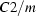

*a* = 19.4755 (10) Å
*b* = 5.6210 (3) Å
*c* = 5.3470 (2) Å
β = 97.222 (3)°
*V* = 580.70 (5) Å^3^
*Z* = 2
Mo *K*α radiation
μ = 0.59 mm^−1^
*T* = 296 K
0.50 × 0.34 × 0.16 mm

#### Data collection

Bruker APEXII CCD diffractometer
Absorption correction: multi-scan (*AX-Scale*; Bruker, 2008 ▶) *T*_min_ = 0.757, *T*_max_ = 0.912
3067 measured reflections
793 independent reflections
694 reflections with *I* > 2s(*I*)
*R*_int_ = 0.028

#### Refinement

*R*[*F* ^2^ > 2σ(*F* ^2^)] = 0.056
*wR*(*F* ^2^) = 0.155
*S* = 1.17
793 reflections
46 parameters
H-atom parameters constrained
Δρ_max_ = 0.47 e Å^−3^
Δρ_min_ = −0.44 e Å^−3^

### 

Data collection: *APEX2* (Bruker, 2008 ▶); cell refinement: *SAINT* (Bruker, 2008 ▶; data reduction: *SAINT*; program(s) used to solve structure: *SHELXS97* (Sheldrick, 2008 ▶); program(s) used to refine structure: *SHELXL97* (Sheldrick, 2008 ▶); molecular graphics: *X-SEED* (Barbour, 2001 ▶) and *Mercury* (Macrae *et al.*, 2006 ▶); software used to prepare material for publication: *publCIF* (Westrip, 2010 ▶) and *PLATON* (Spek, 2009 ▶).

## Supplementary Material

Crystal structure: contains datablocks I, global. DOI: 10.1107/S1600536811012025/bt5503sup1.cif

Structure factors: contains datablocks I. DOI: 10.1107/S1600536811012025/bt5503Isup2.hkl

Additional supplementary materials:  crystallographic information; 3D view; checkCIF report

## Figures and Tables

**Table 1:** Hydrogen-bond geometry (Å, °)

*D*—H⋯*A*	*D*—H	H⋯*A*	*D*⋯*A*	*D*—H⋯*A*
N1—H1*N*⋯O1^i^	0.89	2.35	3.035 (3)	134
N1—H1*N*⋯O1^ii^	0.89	2.35	3.035 (3)	134
N1—H2*N*⋯O1	0.89	2.68	3.435 (4)	143
N1—H2*N*⋯O3	0.89	2.21	3.0308 (14)	153

Symmetry codes: (i) 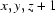; (ii) 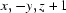.

**Fig. 1.**
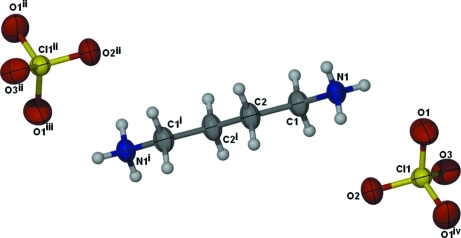
: Molecular structure of the title compound, with atomic numbering scheme and displacement ellipsoids drawn at the 50% probability level.

**Fig. 2.**
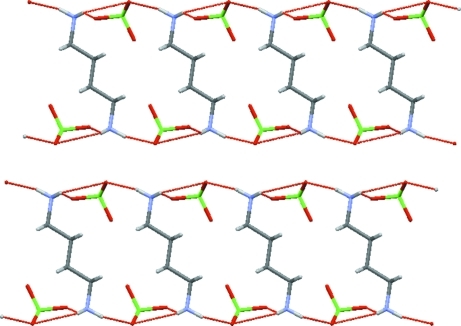
: Packing arrangement of the title compound viewed down the b axis. Hydrogen bonds are indicated by red dashed lines.

**Fig. 3.**
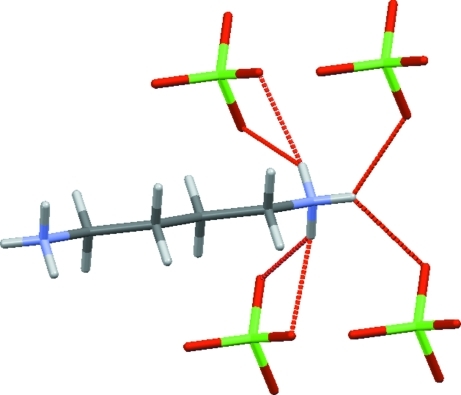
: Close-up view of the title compound clearly showing the bifurcated hydrogen-bonding interactions. Hydrogen bonds are indicated by red dashed lines.

**Fig. 4.**
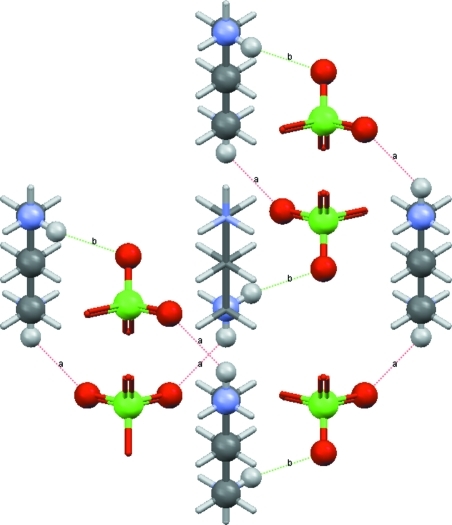
: Close up view of the title compound viewed down the a axis showing the 50-membered level 2 ring motif.

## supplementary crystallographic information

### Comment

The crystal structure of the title compound (I) adds to our current ongoing studies of long-chained diammonium mineral acid salts. Colourless rectangular crystals of butane-1,4-diammonium diperchlorate were synthesized and formed part of our structural chemistry study of the inorganic mineral acid salts of butane-1,4-diamine.

The butane-1,4-diammonium cation lies over an inversion centre and a twofold rotation axis. It also straddles a crystallographic mirror plane. The asymmetric unit contains one-half of a perchlorate anion and one-half of the butane-1,4-diammonium cation. The hydrocarbon chain is also fully extended and is of necessity completely planar as it lies in the crystallographic mirror plane. The molecular structure of (I) is shown in Figure 1.

Figure 2 illustrates the packing arrangement of the title compound (I). Single stacked layers of cations pack together with perchlorate anions inserted between the cation chains in line with the ammonium groups showing a distinct inorganic - organic layering effect that is a common feature of these long-chained diammonium salts. An extensive three-dimensional hydrogen-bonding network is formed.

A close-up view of the hydrogen bonding interactions can be viewed in Figure 3 where very clear evidence of bifurcated interactions can be seen on each hydrogen atom of both ammonium groups. The hydrogen bond distances and angles for (I) can be found in Table 2.

Since the hydrogen bonding network is extremely intricate and complex, we focus on one particularly interesting hydrogen-bonding ring motif in the structure. Figure 4 shows a view of five diammonium cations and five perchlorate anions (viewed down the *a* axis) that are hydrogen bonded together to form a large, level 2, 50-membered ring motif with graph set notation *R*^10^_10_(50). Numerous other ring and chain motifs were identified with *Mercury* (Macrae *et al.*), but since the one in Figure 4 is the highest level motif obtainable in the structure, the other motifs of lower level are not depicted here.

### Experimental

The title compound was prepared by adding butane-1,4-diamine (0.50 g, 5.67 mmol) to 30% perchloric acid (HClO_4_, 2 ml, 9.138 mmol, Merck) in a sample vial. The mixture was then refluxed at 363 K for 2 h. The solution was cooled at 2 K h^-1^ to room temperature. Colourless crystals of butane-1,4-diammonium diperchlorate were collected and a suitable single-crystal was selected for the X-ray diffraction study.

### Refinement

Hydrogen atoms could be identified from the difference Fourier map but once these atoms were refined, their distances from the parent atoms were found to be significantly shorter than the ideal distances for C—H and N—H respectively. The H-atoms were therefore geometrically positioned and refined in the riding-model approximation, with C—H = 0.97 Å, N—H = 0.89 Å, and *U*_iso_(H) = 1.2Ueq(C) or 1.5Ueq(N). The highest peak in the final difference map is 0.69Å from O2 and the deepest hole is 0.69Å from Cl1.

### Crystal data

**Table:**

C_4_H_14_N_2_^2+^·2ClO_4_^−^	*F*(000) = 300
*M**_r_* = 289.07	*D*_x_ = 1.653 Mg m^−^^3^
Monoclinic, *C*2/*m*	Mo *K*α radiation, λ = 0.71073 Å
Hall symbol: -C 2y	Cell parameters from 1622 reflections
*a* = 19.4755 (10) Å	θ = 3.8–28.2°
*b* = 5.6210 (3) Å	µ = 0.59 mm^−^^1^
*c* = 5.3470 (2) Å	*T* = 296 K
β = 97.222 (3)°	Block, colourless
*V* = 580.70 (5) Å^3^	0.50 × 0.34 × 0.16 mm
*Z* = 2	

### Data collection

**Table:**

Bruker APEXII CCD diffractometer	793 independent reflections
Radiation source: fine-focus sealed tube	694 reflections with *I* > 2s(*I*)
graphite	*R*_int_ = 0.028
φ and ω scans	θ_max_ = 28.3°, θ_min_ = 3.8°
Absorption correction: multi-scan (*AX-Scale*; Bruker, 2008)	*h* = −23→25
*T*_min_ = 0.757, *T*_max_ = 0.912	*k* = −7→7
3067 measured reflections	*l* = −6→7

### Refinement

**Table:**

Refinement on *F*^2^	Primary atom site location: structure-invariant direct methods
Least-squares matrix: full	Secondary atom site location: difference Fourier map
*R*[*F*^2^ > 2σ(*F*^2^)] = 0.056	Hydrogen site location: inferred from neighbouring sites
*wR*(*F*^2^) = 0.155	H-atom parameters constrained
*S* = 1.17	*w* = 1/[σ^2^(*F*_o_^2^) + (0.0955*P*)^2^ + 0.3477*P*] where *P* = (*F*_o_^2^ + 2*F*_c_^2^)/3
793 reflections	(Δ/σ)_max_ < 0.001
46 parameters	Δρ_max_ = 0.47 e Å^−^^3^
0 restraints	Δρ_min_ = −0.44 e Å^−^^3^

### Special details

**Table:**

Geometry. All e.s.d.'s (except the e.s.d. in the dihedral angle between two l.s. planes) are estimated using the full covariance matrix. The cell e.s.d.'s are taken into account individually in the estimation of e.s.d.'s in distances, angles and torsion angles; correlations between e.s.d.'s in cell parameters are only used when they are defined by crystal symmetry. An approximate (isotropic) treatment of cell e.s.d.'s is used for estimating e.s.d.'s involving l.s. planes.
Refinement. Refinement of *F*^2^ against ALL reflections. The weighted *R*-factor *wR* and goodness of fit *S* are based on *F*^2^, conventional *R*-factors *R* are based on *F*, with *F* set to zero for negative *F*^2^. The threshold expression of *F*^2^ > σ(*F*^2^) is used only for calculating *R*-factors(gt) *etc*. and is not relevant to the choice of reflections for refinement. *R*-factors based on *F*^2^ are statistically about twice as large as those based on *F*, and *R*- factors based on ALL data will be even larger.

### Fractional atomic coordinates and isotropic or equivalent isotropic displacement parameters (Å^2^)

**Table:**

	*x*	*y*	*z*	*U*_iso_*/*U*_eq_	
C1	0.57860 (17)	0.0000	0.7538 (6)	0.0466 (8)	
H1	0.5661	0.1394	0.8450	0.056*	
C2	0.53822 (16)	0.0000	0.4976 (6)	0.0467 (8)	
H2	0.5508	−0.1394	0.4065	0.056*	
Cl1	0.65841 (4)	0.5000	0.27016 (13)	0.0403 (3)	
N1	0.65459 (14)	0.0000	0.7476 (5)	0.0450 (7)	
H1N	0.6762	0.0000	0.9045	0.068*	
H2N	0.6666	0.1293	0.6673	0.068*	
O1	0.69616 (13)	0.2898 (5)	0.2214 (5)	0.0755 (7)	
O2	0.59205 (16)	0.5000	0.1238 (6)	0.0698 (9)	
O3	0.65031 (17)	0.5000	0.5341 (5)	0.0633 (8)	

### Atomic displacement parameters (Å^2^)

**Table:**

	*U*^11^	*U*^22^	*U*^33^	*U*^12^	*U*^13^	*U*^23^
C1	0.0461 (18)	0.061 (2)	0.0321 (15)	0.000	0.0021 (12)	0.000
C2	0.0403 (18)	0.068 (2)	0.0315 (15)	0.000	0.0028 (12)	0.000
Cl1	0.0480 (5)	0.0380 (5)	0.0344 (5)	0.000	0.0031 (3)	0.000
N1	0.0425 (15)	0.0501 (17)	0.0398 (14)	0.000	−0.0049 (11)	0.000
O1	0.0818 (14)	0.0697 (16)	0.0730 (14)	0.0253 (12)	0.0014 (11)	−0.0236 (11)
O2	0.0619 (18)	0.069 (2)	0.071 (2)	0.000	−0.0187 (14)	0.000
O3	0.095 (2)	0.0596 (17)	0.0377 (14)	0.000	0.0171 (13)	0.000

### Geometric parameters (Å, °)

**Table:**

C1—N1	1.484 (4)	Cl1—O1	1.433 (2)
C1—C2	1.492 (4)	Cl1—O1^ii^	1.433 (2)
C1—H1	0.9700	Cl1—O3	1.440 (3)
C2—C2^i^	1.492 (6)	N1—H1N	0.8900
C2—H2	0.9700	N1—H2N	0.8900
Cl1—O2	1.424 (3)		
			
N1—C1—C2	113.0 (3)	O2—Cl1—O1^ii^	110.54 (12)
N1—C1—H1	109.0	O1—Cl1—O1^ii^	111.1 (2)
C2—C1—H1	109.0	O2—Cl1—O3	109.6 (2)
H1—C1—H1^iii^	107.8	O1—Cl1—O3	107.48 (13)
C1—C2—C2^i^	113.3 (3)	O1^ii^—Cl1—O3	107.48 (13)
C1—C2—H2	108.9	C1—N1—H1N	109.5
C2^i^—C2—H2	108.9	C1—N1—H2N	109.5
H2—C2—H2^iii^	107.7	H1N—N1—H2N	109.5
O2—Cl1—O1	110.54 (12)		
			
N1—C1—C2—C2^i^	180.0		

Symmetry codes: (i) −*x*+1, −*y*, −*z*+1; (ii) *x*, −*y*+1, *z*; (iii) *x*, −*y*, *z*.

### Hydrogen-bond geometry (Å, °)

**Table:**

*D*—H···*A*	*D*—H	H···*A*	*D*···*A*	*D*—H···*A*
N1—H1N···O1^iv^	0.89	2.35	3.035 (3)	134
N1—H1N···O1^v^	0.89	2.35	3.035 (3)	134
N1—H2N···O1	0.89	2.68	3.435 (4)	143
N1—H2N···O3	0.89	2.21	3.0308 (14)	153

Symmetry codes: (iv) *x*, *y*, *z*+1; (v) *x*, −*y*, *z*+1.
